# Associations Between Six Core Processes of Psychological Flexibility and Functioning for Chronic Pain Patients: A Three-Level Meta-Analysis

**DOI:** 10.3389/fpsyt.2022.893150

**Published:** 2022-07-11

**Authors:** Dongyan Ding, Mengna Zheng

**Affiliations:** School of Educational Science, Anhui Normal University, Wuhu, China

**Keywords:** processes of psychological flexibility, physical functioning, psychological functioning, chronic pain, meta-analysis, acceptance, acceptance and commitment therapy

## Abstract

The previous research showed contradictions in the relationships between psychological flexibility processes and functioning. This meta-analysis is the first to provide a comprehensive meta-analysis of the associations between six core processes of psychological flexibility and functioning among chronic pain patients. Four databases were searched (PsycINFO; PubMed; CINAHL; Web of Science) along with reference lists. Thirty-six cross-sectional studies were included (7,812 chronic pain patients). A three-level meta-analytic model was used to examine the associations. The publication bias was assessed with the Egger test, funnel plot, and *p*-curve analysis. Significant associations were found between functioning and six processes of psychological flexibility (i.e., acceptance, defusion, present moment, committed action, self as context, and values). Except for the relationship between defusion and functioning, the relationships between the other five psychological flexibility processes and functioning were all moderated by domains of functioning. No moderators were found regarding age, percentage of females, country, or type of instrument used to measure functioning. These findings may carry significant implications for chronic pain patients and clinical workers. It might be more effective to focus on functioning-related psychological flexibility processes rather than all therapy packages if the relationships between functioning and specific processes of psychological flexibility were better informed. Limitations were also discussed.

## Introduction

Chronic pain is one of the most common physical problems all over the world in the general population (1–3) and is a source of distress and disability that affects all aspects of a patient’s life (4, 5). Furthermore, individuals in a state of psychological distress experience more intense pain, leading to a reciprocal reinforcement between psychological distress and pain (5, 6). Chronic pain also costs economically higher than other diseases (4), leading to immense suffering for their families and high costs on our communities and healthcare systems (7).

Traditional pain management has been focused mainly on reducing pain and pain-related distress, with pain interference (i.e., functional impairment) being a neglected dimension (8). In treating chronic pain, recent research supports the view that a critical issue concerns the changes necessary to improve physical and psychological functioning (8, 9). One of these views comes from acceptance and commitment therapy (ACT), which defends a greater focus on functioning, and encourages patients to engage with valued activities and meaningful areas even when pain and distress persist (9, 10).

The expressed goal of ACT is to increase behaviors in the direction of functionality by increasing psychological flexibility (11, 12). Psychological flexibility refers to an individual’s ability to focus on the present moment, move toward their goals, and persist or change behaviors to serve valued ends (13–15). Actually, the psychological flexibility model of ACT can be seen as a basis for an integrated and progressive psychological approach to chronic pain management (16). This model fully integrates cognitive and environmental influences as the core processes of healthy and problem behaviors (16). As suggested by the psychological flexibility model, pain and suffering are inherent aspects of human life, and the psychological function of pain is central to the analysis (8). It means that a behavioral response is not directly related to the level of pain intensity but rather to its function or meaning for the individual in that particular context (8). Thus, individual functional behavior can be increased by improving psychological flexibility. Actually, many researchers suggested that ACT is more effective than controls (except CBT) in improving functional impairment or increasing values-congruent behaviors (17–19).

The previous studies with chronic pain patients have supported the role of the various components of psychological flexibility in reducing disability and functional impairment (20, 21). However, different results also appeared in different studies for the exact relationship between outcome variables. For example, some studies showed that the magnitude of the correlation coefficient between acceptance and functioning was small (22) or medium (23, 24), while in other studies, the effect sizes were large (25, 26). These discrepancies can also be found in the relationship between functioning and other psychological flexibility processes (27, 28). It is worth noting that most studies examining the relationship between psychological flexibility and functioning take psychological flexibility or functioning as a whole. However, the psychological flexibility model is comprised of six core ACT processes, i.e., acceptance, defusion, present moment, self as context, committed action, and values (15). All these six components may have a particular relationship with the functioning of patients with chronic pain. Specifically, *acceptance* is defined as acknowledging and experiencing unwanted thoughts and feelings without having to follow, reduce, or alter them and has been linked to better functioning in chronic pain patients (29, 30). For chronic pain patients, *defusion* involves learning to distance themself from pain and distress in order to reduce the influence of these experiences on behavior. The *present moment* entails flexible awareness and non-judgmental contact with ongoing events. *Self as context* entails an experience of taking a perspective from which to observe one’s psychological experiences without attachment to them or an investment in which particular experiences occur. *Values* are chosen qualities of purposive action that we want to achieve and reflect in our behavior. *Committed action* is the ability to flexibly persist in actions guided by values (15, 31). These six core processes can be fostered in the ACT by different exercises. From the view of the psychological flexibility model, chronic pain patients can relieve the psychological burden or improve their psychological functioning through accepting inner experiences, being mindful, and participating in actions that are aligned with individual goals and values (2, 32). Likewise, many researches have classified functioning into physical and psychological functioning (7, 33). Physical functioning is made up of independent ambulation, mobility, and body care and movement scales, while the psychosocial domain is made up of social interaction, alertness, emotional behavior, and communication (7, 33).

To date, no study makes a comprehensive meta-analysis of the relationship between specific mechanisms of psychological flexibility (e.g., acceptance, defusion, present moment, self as context, committed action, and values) and different domains of functioning. Many current researches examined the relationship between psychological flexibility and function without considering their sub-domains. Some researchers thought it is necessary to find which components of therapy work for which type of patient on which outcome/s and try to understand why (34). It would be hard to understand the mechanisms of psychological flexibility for functioning if we take psychological flexibility as a whole. The science and core clinical competencies of ACT also require the understanding of process-based therapy, which refers to contextually specific evidence-based processes associated with evidence-based procedures (35, 36). And the call for process-based therapy suggested that focusing on specific change processes could provide evidence-based methods and make the therapies person-centered to enhance particular people’s physical and psychological health more efficiently (35, 36). A meta-analysis of this subject is essential to understand the basic psychological processes underlying the functioning, which would consequentially form the basis for more robust testing of causal and manipulable relationships. Suppose we knew which process of psychological flexibility is more closely related to the domains of functioning. In that case, we could provide targeted intervention services to chronic pain patients to improve their functioning. Thus, it may have important implications for healthcare professionals, organizations, and patient care.

As suggested by the psychological flexibility model, increased psychological flexibility is not intended to reduce pain intensity, while the psychological function of pain is central to the analysis (8). Therefore, we hypothesize that the components of psychological flexibility may be more relevant to psychological functioning than to physical functioning. Besides, some studies suggested associations between psychological factors and functioning may be influenced by culture (37). Hence, we assumed that culture might be a moderating variable. We also considered age and the proportion of females as moderators.

The primary aim of this review was to identify and integrate all published findings on associations between different processes of psychological flexibility and domains of functioning, and address an analytic question about the magnitude and direction of the associations among chronic pain patients. A second aim is to determine which variables potentially moderate the relationships. We hypothesized that the following five moderators would systematically influence the effect: (1) the domain of functioning, (2) the age of the target sample, (3) the country, (4) the proportion of females, and (5) the type of measurements of functioning. A third research goal is to address descriptive questions about how these variables are being measured for chronic pain.

## Materials and Methods

### Selection of Studies

The meta-analysis was reported following the Preferred Reporting Items for Systematic Reviews and Meta-analyses (PRISMA) statement (38).

The first author conducted a search using PsycINFO, PubMed, CINAHL, and Web of Science, all of which were searched on October 1, 2021, and updated on May 27, 2022. No date restrictions were applied to the search to maximize the search strategy. Because acceptance and value are wide-ranging, this study mainly uses instruments that measure them instead of these constructs. Other instruments commonly used to measure psychological flexibility processes were also used in order to minimize potential publication bias. The main search terms used included keywords and free words: [(Acceptance Questionnaire) OR (Valued Living Questionnaire) OR (Chronic Pain Values Inventory) OR (Valuing Questionnaire) OR (Personal Values Questionnaire) OR (Mindful Attention Awareness Scale) OR (Mindfulness) OR (Avoidance and Fusion Questionnaire) OR (Thought Suppression Inventory) OR (Automatic Thought Questionnaire) OR (present moment) OR (committed action) OR (self-as-context) OR (cognitive defusion) OR (psychological inflexibility) OR (psychological flexibility)] AND (functioning OR dysfunction OR (pain disability) OR (pain interference)] AND [(chronic pain) OR fibromyalgia). In addition, reference lists of eligible studies and relevant review articles, as well as relevant meta-analyses were manually searched to minimize potential publication bias.

#### Inclusion Criteria

(a) The sample population included chronic pain patients and fibromyalgia patients;
(b) One of six core processes of psychological flexibility was measured as well as the functioning (i.e., psychological functioning and/or physical functioning) of the patients.
(c) The relationship between processes of psychological flexibility and functioning was reported with Pearson’s *r* correlation coefficient.

#### Exclusion Criteria

(a) Review, meta-analysis, or theoretical articles;
(b) Without reporting Pearson’s *r* correlation coefficient.

Difficulties in deciding the selection were discussed between the two authors. According to the criteria, any ambiguity about studying eligibility was settled *via* discussion, and a full consensus was reached between the two authors.

### Data Extraction and Coding

Data extraction was performed by the first author and checked by the second author. If there were disagreements, agreements would be reached through a full consultation. Extracted data include: authors and year of publication, country, instruments used to measure processes of psychological flexibility and functioning, study characteristics (e.g., sample size, mean age, and percentage of females), and effect sizes.

According to the authors’ definition, processes flexibility was coded as belonging to the six dimensions, i.e., acceptance, defusion, present moment, self as context, committed action, and values. Functioning was coded as three domains, i.e., psychological, physical, and overall functioning. When the total functioning score was used, and psychological or physical functioning was not reported, it would then be classified under the “overall functioning” heading. Besides psychological functioning, emotional and social functioning were also coded as psychological functioning. Physical functioning was coded as physical functioning.

We created three dummy variables for domains of functioning: psychological functioning, physical functioning, and overall functioning. The value 1 in these dummy variables is indicative of the specific type of functioning being applicable, whereas the value 0 indicates that the specific type of functioning is not applicable. We also created dummy variables for the type of the measurements of functioning. If the measurement was used only in one effect size, it would be coded as “other” to reduce the number of dummy variables. These dummy variables are mutually exclusive. Directions of these effects were adjusted accordingly within each study. For example, the direction of the relationship between acceptance and dysfunction would be reversely coded to represent the relationship between acceptance and functioning.

### Data Analysis

In the present study, a three-level meta-analytic model was used to synthesize effect sizes and conduct moderator analyses to achieve maximum statistical power (39). The three-level model examined three sources of variance: sampling variance of the observed effect sizes (Level 1); variance between effect sizes from the same study (Level 2); and variance between studies (Level 3) (39, 40). Some scholars have noted that heterogeneity can be considered substantial if less than 75% of the total variance can be attributed to level 1 (41). Therefore, potential moderating effects that may impact the overall effect will be examined according to the 75% rule.

When a study reported multiple effect sizes due to the multiple instruments used to assess the same construct, all relevant effect sizes would be extracted so that we could control for within-study dependency without reducing the number of effect sizes available in the literature (39). All analyses were conducted in R version 4.1.2 (42), using the meta and metafor package (39). The R syntax was written following related tutorials (41, 43). All model parameters were estimated using the restricted maximum likelihood method.

Due to differences in measurement tools, the effect sizes were analyzed using the random-effects model. Individual study effect sizes (*r* and *r*s) and sample sizes were entered to calculate pooled effect size estimates (*r*). All extracted effects were converted to Fisher’s *Z-*values and weighted by sample size before analysis. These effects were then meta-analyzed, and the results were subsequently converted back to correlations for interpretation (44). In accordance with Cohen’s convention, the magnitude of effect for *r* is classified as small (0.10), medium (0.30), or large (0.50) with 95% CI.

The heterogeneity among the results was tested by the Q test and the *I*^2^ test (45, 46). If *I*^2^ > 50%, it is considered to have moderate-to-high heterogeneity. Egger test and funnel plot assessed the possibility of publication bias, with significant publication bias as *p* < 0.1 (45). When it revealed possible publication bias, *trim and fill* analyses were performed to provide an adjusted average effect size (47) to correct. *P*-curve analysis was also used to detect selective reporting (48, 49). The *p*-curve method is based on the distribution of significant *p*-values of a set of findings. If an actual effect exists, it will skew to the right or the left if selective reporting is prevalent (48, 49).

## Results

### Description of Studies

#### Studies Characteristics

**FIGURE 1:**
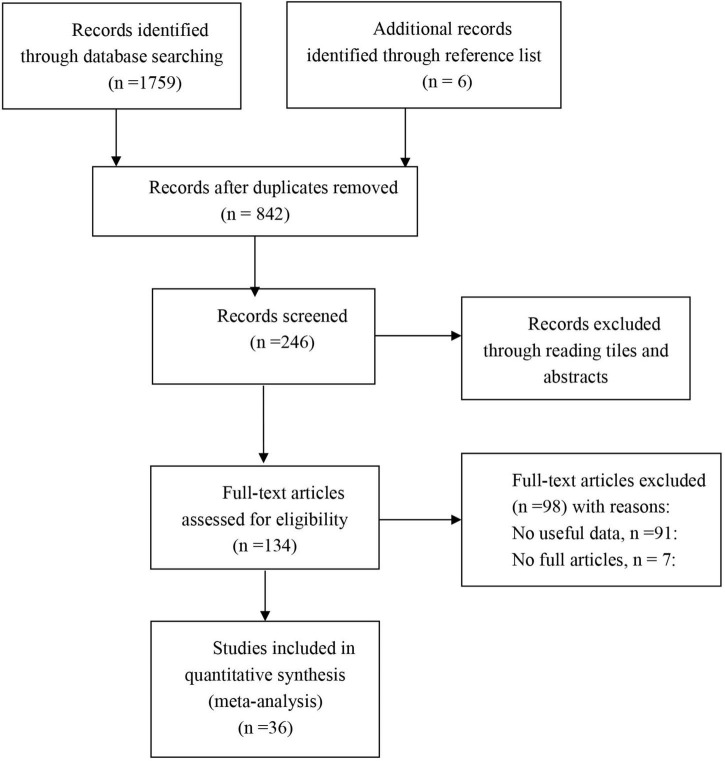
Study flow diagram.

Initially, 1,759 citations were identified through searches of electronic bibliographic databases and reference lists. After detecting duplicates and screening titles and abstracts for relevance, 134 articles were identified as potentially eligible for further assessment. After reading the full text of each article, 36 studies met the criteria and were included in this study (see Figure 1 for the details).

The characteristics of the 36 included studies were summarized in Table 1, from which a total of 109 correlations could be extracted. Sample sizes for included studies ranged from 23 to 555 (total participants = 7,812). Among them, 15 studies were from the United Kingdom, six studies from the United States, four from Spain, Australia, and Sweden, one from Portugal, Singapore, Belgium, Republic of Cyprus, and multi-country (i.e., United States (30.1%), Ireland (30.1%), England (21.13%)]. The mean age of participants in these studies ranged from 14.44 to 69.3. There were 763 adolescents, and most participants were adults (97.67%). The proportion of females ranged from 39.2 to 100%.

**TABLE 1:** Study characteristics and effect size.

References	Country	Mean age	% female	*N*	Process of PF	Measure(s) of processes of PF	Measure(s) of functioning	*r*
Åkerblom et al. (67)	Sweden	41	72.1	462	Committed action	CAQ-18	SF-36	0.2
					Committed action	CAQ-8	SF-36	0.16
					Committed action	CAQ-8	SF-36	0.3
					Committed action	CAQ-18	SF-36	0.28
Åkerblom et al. (68)	Sweden	41	71.1	315	Committed action	CAQ	MPI-pain interference	0.26
					Acceptance	CPAQ	MPI-pain interference	0.61
					Values	CPVI	MPI-pain interference	0.27
					Defusion	PIPS	MPI-pain interference	0.43
Beeckman et al. (69)	Belgium	13.76	61.02	59	Defusion	AFQ-Y	Pediatric quality of life inventory	0.43
					Acceptance	CPAQ-Adolescent	Pediatric quality of life inventory	0.45
Carriere et al. (70)	USA	47.5	67	354	Acceptance	CPAQ-8	PROMIS physical functioning item bank	0.5
Carvalho et al. (71)	Portugal	50.49	100	49	Values	VQ	PDI	0.13
Catala et al. (72)	Spain	55.91	100	228	Defusion	CFQ	FIQ	0.29
Cebolla et al. (73)	Spain	52.4	96	251	Present moment	MAAS	FIQ	0.46
Feinstein et al. (23)	United States	15	91	23	Defusion	AFQ-Y	FDI	0.35
					Acceptance	CPAQ-Adolescent	FDI	0.38
Fish et al. (74)	United States, Ireland, England	53.07	79.54	535	Defusion	PIPS	BPI	0.27
					Acceptance	CPAQ-8 activity engagement	BPI	0.5
					Acceptance	CPAQ-8 pain willingness	BPI	0.34
Foote et al. (24)	United States	41.5	88.2	103	Values	CPVI	MIDAS	0.47
					Acceptance	CPAQ	MIDAS	0.35
Galán et al. (57)	Spain	47.21	91.9	258	Committed action	CAQ-8	PDI	0.35
Gauntlett-Gilbert et al. (75)	United Kingdom	15.33	71.28	346	Acceptance	CPAQ-A8	BAPQ	0.53
					Acceptance	CPAQ-A8	BAPQ	0.38
					Acceptance	CPAQ-A (full length)	BAPQ	0.52
					Acceptance	CPAQ-A (full length)	BAPQ	0.35
Gentili et al. (76)	Sweden	47.4	81	252	Values	VQ	PII	0.38
Graham et al. (77)	United Kingdom	46.74	58.39	137	Defusion	CFQ	HAQ-DI	0.06
					Values	ELS	HAQ-DI	−0.03
Kanzler, et al. (78)	United States	NA	42	207	Acceptance	CPAQ	Oswestry Disability Index (ODI)	0.63
McCracken and Zhao-O’Brien (79)	United Kingdom	42.4	63.9	144	Acceptance	CPAQ	SIP-physical disability	0.49
					Acceptance	CPAQ	SIP-psychological disability	0.49
McCracken and Jones (80)	United Kingdom	64.3	62.5	40	Present moment	MAAS	SIP-physical disability	0.49
					Present moment	MAAS	SIP-psychological disability	0.55
					Values	CPVI	SIP-physical disability	−0.06
					Values	CPVI	SIP-psychological disability	0.19
					Acceptance	CPAQ	SIP-physical disability	0.55
					Acceptance	CPAQ	SIP-psychological disability	0.59
McCracken and Velleman (21)	United Kingdom	61.5	58.2	239	Present moment	MAAS	SF-36-physical disability	0.04
					Present moment	MAAS	SF-36-emotional functioning	0.48
					Present moment	MAAS	SF-36-social functioning	0.37
					Values	CPVI	SF-36-physical disability	0.36
					Values	CPVI	SF-36-emotional functioning	0.45
					Values	CPVI	SF-36-social functioning	0.53
					Acceptance	CPAQ	SF-36-physical disability	0.41
					Acceptance	CPAQ	SF-36-emotional functioning	0.51
					Acceptance	CPAQ	SF-36-social functioning	0.55
McCracken and Vowles (81)	United Kingdom	48.1	56.5	115	Acceptance	CPAQ	SIP-physical disability	0.25
					Values	CPVI	SIP-physical disability	0.37
					Values	CPVI	SIP-psychological disability	0.39
					Acceptance	CPAQ	SIP-psychological disability	0.4
McCracken et al. (22)	United Kingdom	43.8	63	159	Values	CPVI	SIP	0.33
					Present moment	MAAS	SIP	0.1
					Acceptance	CPAQ	SIP	0.28
McCracken et al. (59)	United Kingdom	43	69.3	150	Self as context	EQ	SIP-physical disability	−0.02
					Self as context	EQ	SIP-psychological disability	0.47
					Acceptance	CPAQ	SIP-physical disability	0.2
					Present moment	MAAS	SIP-physical disability	0.03
					Present moment	MAAS	SIP-psychological disability	0.56
					Values	CPVI	SIP-physical disability	0.24
					Values	CPVI	SIP-psychological disability	0.49
					Acceptance	CPAQ	SIP-psychological disability	0.51
McCracken et al. (20)	United Kingdom	47.3	66.9	352	Self as context	EQ	SF-36	0.01
					Self as context	EQ	SF-36	0.37
					Self as context	EQ	SF-36	0.04
					Self as context	EQ	SF-36	0.32
Nigol and Di Benedetto (82)	Australia	49.54	83.16	190	Present moment	FFMQ	Brief Pain Inventory (BPI)	0.32
					Self as context	FFMQ	Brief Pain Inventory (BPI)	0.45
					Defusion	FFMQ	Brief Pain Inventory (BPI)	0.35
Scott et al. (27)	United Kingdom	69.3	61.7	60	Acceptance	CPAQ	SF-36-physical disability	0.32
					Acceptance	CPAQ	SF-36-social functioning	0.2
					Defusion	CFQ	SF-36-physical disability	0.02
					Defusion	CFQ	SF-36-social functioning	0.21
					Committed action	CAQ	SF-36-physical disability	0.27
					Committed action	CAQ	SF-36-social functioning	0.25
					Self as context	EQ	SF-36-physical disability	−0.09
					Self as context	EQ	SF-36-social functioning	0.01
Scott et al. (55)	United Kingdom	45.22	68.3	294	Acceptance	CPAQ-8	Brief Pain Inventory (BPI)	0.32
Solé et al. (83)	Spain	14.44	61	281	Defusion	CFQ	FDI	0.3
Trainor et al. (84)	Australia	46	95	337	Acceptance	BEAQ	Fibromyalgia Impact Questionnaire	0.52
Vasiliou et al. (85)	Republic of Cyprus	57.08	81.6	160	Committed action	CPAQ20	Brief pain inventory (BPI)	0.41
					Committed action	CPAQ8	Brief Pain Inventory (BPI)	0.42
Waldron et al. (61)	United Kingdom	14.6	72	54	Acceptance	CPAQ-activity engagement	BAPQ	0.4
					Acceptance	CPAQ-activity engagement	BAPQ	0.34
					Acceptance	CPAQ -pain willingness	BAPQ	0.19
					Acceptance	CPAQ -pain willingness	BAPQ	0.2
					Present moment	CAMM	BAPQ	0.17
					Present moment	CAMM	BAPQ	0.11
Williams and Cano (25)	United States	58.84	47.1	51	Present moment	FFMQ-acting with awareness	MPI-pain interference	0.31
					Self as context	FFMQ-non-judging	MPI-pain interference	0.27
					Defusion	FFMQ-non-reactivity	MPI-pain interference	−0.05
					Acceptance	CPAQ	MPI-pain interference	0.8
					Defusion	FFMQ-non-judging	MPI-pain interference	0.27
Wong et al. (86)	United States	48.2	39.2	97	Acceptance	PIPS	WHYMPI	0.4
					Defusion	PIPS	WHYMPI	0.24
Yang et al. (87)	Singapore	45.27	56	200	Committed action	CAQ	BPI	0.26
					Acceptance	CPAQ-8	BPI	0.69
Yu et al. (28)	United Kingdom	44.73	93.3	298	Self as context	SEQ	BPI	0.26
Yu et al. (26)	United Kingdom	42.97	72.7	89	Defusion	CFQ-7	BPI	0.37
					Defusion	CFQ-7	WSAS	0.35
					Committed action	CAQ-8	BPI	0.36
					Committed action	CAQ-8	WSAS	0.4
					Acceptance	CPAQ-8	BPI	0.23
					Acceptance	CPAQ-8	WSAS	0.42
Yu et al. (31)	United Kingdom	40	86.3	555	Self as context	SEQ-8	WSAS	0.68
					Committed action	CAQ-8	WSAS	0.67
					Acceptance	CPAQ-8	WSAS	0.61
Zetterqvist et al. (88)	Sweden	48.7	75	368	Acceptance	PIPS	PDI	0.51
					Defusion	PIPS	PDI	0.19

*r, the correlation coefficient between processes of psychological flexibility and functioning; N, the total sample size; FFMQ, Five facet mindfulness questionnaire; CPAQ, chronic pain acceptance questionnaire; MAAS, mindful attention awareness scale; PIPS, psychological Inflexibility in pain scale; MAAS, mindful attention awareness scale; BEAQ, brief experiential avoidance questionnaire; CFQ, cognitive fusion questionnaire; CPVI, chronic pain values inventory; CAQ, committed action questionnaire; SEQ, self experiences questionnaire; CAMM, child and adolescent mindfulness measure; EQ, experiences questionnaire; AFQ-Y, adolescents completed the avoidance and fusion questionnaire for youth; VQ, valuing questionnaire; ELS, the engaged living scale; MPI, multidimensional pain inventory; PDI, pain disability index; WSAS, work and social adjustment scale; BPI, brief pain inventory; SIP, sickness impact profile; SF-36, short-form health survey; FDI, functional disability inventory; BAPQ, bath adolescent pain questionnaire; HAQ-DI, the Stanford health assessment questionnaire-disability index; FIQ, fibromyalgia impact questionnaire; WSAS, work and social adjustment scale; MIDAS, migraine disability assessment scale; PII, pain interference index; WHYMPI, West Haven-Yale multidimensional pain inventory.*

#### The Measurements

Acceptance was measured with the Chronic Pain Acceptance Questionnaire (CPAQ) and Psychological Inflexibility in Pain Scale (PIPS). Committed action was measured with the Committed Action Questionnaire (CAQ). Cognitive defusion was measured with the Cognitive fusion questionnaire (CFQ), subscales of PIPS, the Avoidance and Fusion Questionnaire for Youth (AFQ-Y), and subscales of the Five Facet Mindfulness Questionnaire (FFMQ). The present moment was measured with the Mindful attention awareness scale (MAAS), the Child and Adolescent Mindfulness Measure (CAMM), and subscales of FFMQ (i.e., acting with awareness). Self as context was measured with Experiences Questionnaire (EQ), Self-experiences questionnaire (SEQ), and FFMQ-non-judgment (50). Values were measured with the Chronic Pain Values Inventory (CPVI), the Valuing Questionnaire (VQ), and the Engaged living scale (ELS).

Functional impairment mainly was measured with the Brief Pain Inventory-functional impairment subscale (BPI), Sickness Impact Profile (SIP), Short-Form Health Survey (SF-36), Pain Disability Index (PDI), Functional Disability Inventory (FDI), Fibromyalgia impact questionnaire (FIQ), and Multidimensional Pain Inventory (MPI).

### Meta-Analyses

#### Acceptance and Functioning

**FIGURE 2:**
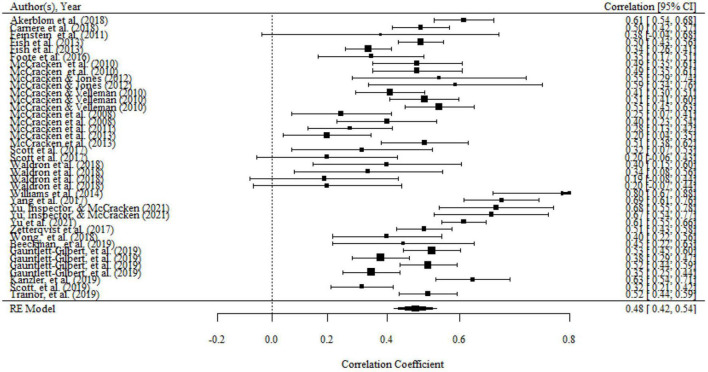
Forest plot of effect size (*r*) for the relationship between acceptance and functioning.

Aggregating across 39 correlations in 24 studies that examined the relationship between acceptance and functioning, the overall effect size was statistically significant and medium to large (*r* = 0.48, 95% CI = 0.42, 0.54, *p* < 0.001; *I^2^* = 83.89%, *Q* = 198.83, *df* = 38, *p* < 0.001). The results were presented in a forest plot in Figure 2.

There were significant variances within the same studies (i.e., level 2 variance) and between studies (i.e., level 3 variance). The details can be seen in Table 2. Therefore, moderator analyses were conducted in order to determine variables that can explain level 2 and level 3 variance. Moderating effects of mean age, percentage of females, country, domains of functioning, and type of measurements of functioning have been evaluated separately in univariate models. We found a significant moderating effect of the domains of functioning on the association, as shown by the results of the omnibus test (*F*_(2,36)_ = 6.63, *p* < 0.01). The mean effect of association between acceptance and overall functioning (*r* = 0.59, 95% CI = 0.49, 0.69, *p* < 0.001) and psychological functioning (*r* = 0.52, 95% CI = 0.40, 0.64, *p* < 0.001) were both significantly larger than that association of acceptance and physical functioning (*r* = 0.38, 95% CI = 0.22, 0.53, *p* < 0.01). However, there was no significant difference in the association between acceptance and psychological functioning and overall functioning. No significant moderating effects were found for the percentage of females, mean age, country, and type of functioning measurements.

**TABLE 2:** Results for the overall mean effect sizes of the relationship between six processes and functioning.

Processes of PF	# Studies	# ES	Mean r	95% CI	% var. at level 1	Level 2 variance	% Var. at level 2	Level 3 variance	% Var. at level 3
Acceptance	24	39	0.48	0.42, 0.54	13.99	0.01*	25.89	0.02**	60.12
Committed action	8	14	0.32	0.26, 0.39	37.39	0.00	13.31	0.01	49.30
Defusion	13	16	0.27	0.20, 0.34	42.43	0.00	0.00	0.01	57.57
Present moment	8	13	0.31	0.19, 0.43	15.71	0.04**	85.27	0.00	0.00
Self as context	7	12	0.21	0.08, 0.33	11.12	0.03**	88.88	0.00	0.00
Values	10	15	0.31	0.20, 0.41	20.43	0.01	31.23	0.02	48.34

PF, psychological flexibility; # Studies, number of studies; # ES, number of effect sizes; CI, confidence interval; Sig, significance; Mean r, Mean effect size expressed as a Pearson’s correlation; Var, variance; Level 1 variance, sampling variance of observed effect sizes; Level 2 variance, variance between effect sizes extracted from the same study; Level 3 variance, variance between studies; *p < 0.05; **p < 0.01.

**FIGURE 3:**
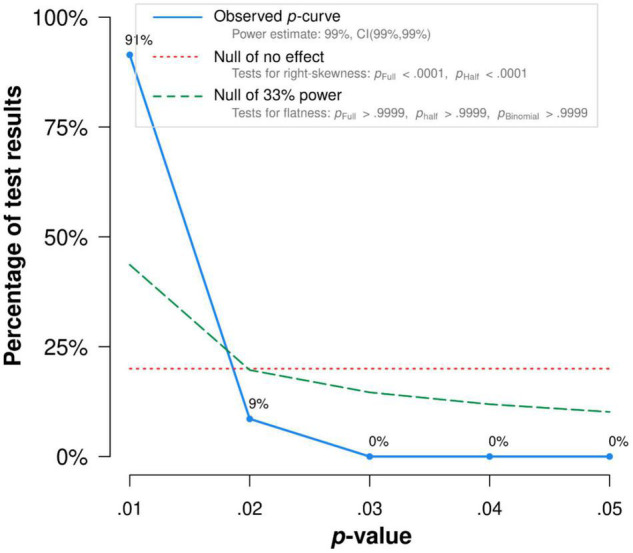
The *p*-Curve for statistically significant results on the relationship between acceptance and functioning. The observed *p*-curve includes 35 statistically significant (*p* < 0.05 results, of which 35 are *p* < 0.025. There were four additional results entered but excluded from *p*-curve because they were *p* > 0.05.

There was no publication bias in Egger tests and Funnel plot (*p* > 0.1) on the relationship between acceptance and functioning. Both the full *p*-curve and the half *p*-curve test were significant with *p* < 0.0001 (*Z* = −31.74, *Z* = −30.62), which indicated that the distribution of *p*-values is significant right-skewed, as seen in Figure 3. Hence, the results further support the initial assessment that evidential value is present in the literature.

#### Committed Action and Functioning

**FIGURE 4:**
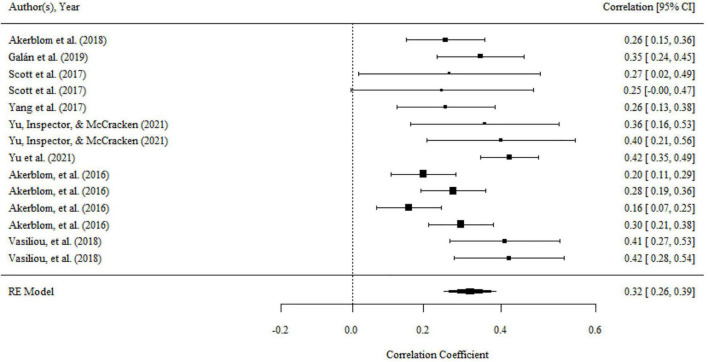
Forest plot of effect size (*r*) for the relationship between committed action and functioning.

Aggregating across 14 correlations that examined the relationship between committed action and functioning, the overall effect size was statistically significant and medium (*r* = 0.32, 95% CI = 0.26, 0.39, *p* < 0.001; *I^2^* = 61.20%, *Q* = 35.04, *df* = 14, *p* < 0.01). The results were presented in a forest plot in Figure 4.

There were no significant variances within level 2 and level 3. However, the variance within level 1 was less than 75% (i.e., 37.39), then moderator analyses were conducted. We found a significant moderating effect of the domains of functioning on the association, as shown by the results of the omnibus test (*F*_(2,11)_ = 4.01, *p* < 0.05). The relationship of committed action with overall functioning (*r* = 0.38, 95% CI = 0.31, 0.45, *p* < 0.001) was significantly larger than that with psychological functioning (*r* = 0.24, 95% CI = 0.13, 0.34, *p* < 0.001). but there was no significant difference between the mean effect of overall functioning and physical functioning (*r* = 0.25, 95% CI = 0.14, 0.35, *p* < 0.001). The domain of functioning was the main source of heterogeneity. After it was included to analysis, the heterogeneity was significantly reduced (*Q* = 17.32, *df* = 11, *p* > 0.05).

**FIGURE 5:**
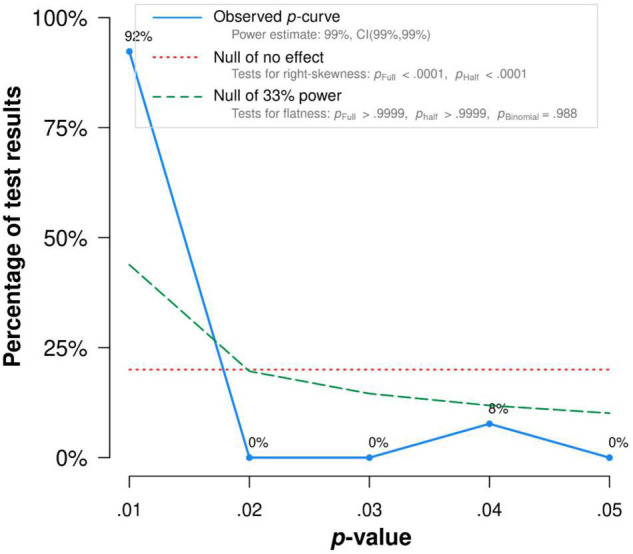
The *p*-Curve for statistically significant results on the relationship between committed action and functioning. The observed *p*-curve includes 13 statistically significant (*p* < 0.05) results, of which 12 are *p* < 0.025. There was one additional result entered but excluded from *p*-curve because it was *p* > 0.05.

There was no publication bias in Egger tests and Funnel plot (*p* > 0.1) on the relationship between acceptance and functioning. Both the full *p*-curve and the half *p*-curve test were significant with *p* < 0.0001 (*Z* = −14.19, *Z* = −14.29), which indicated that the distribution of *p*-values is significant right-skewed, as seen in Figure 5. Hence, the results further support the initial assessment that evidential value was present in the literature.

#### Defusion and Functioning

**FIGURE 6:**
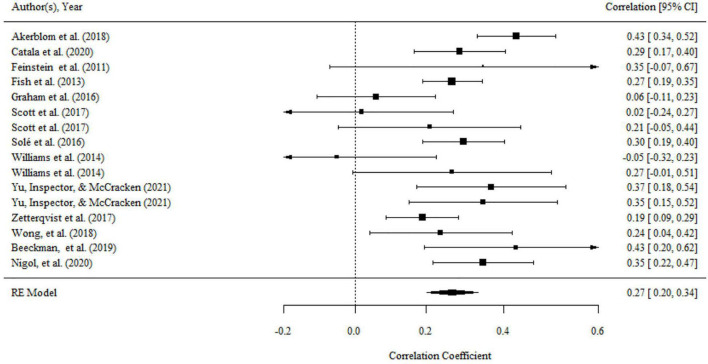
Forest plot of effect size (*r*) for the relationship between defusion and functioning.

Aggregating across 16 correlations in 13 studies that examined the relationship between defusion and functioning, the overall effect size was statistically significant and nearly medium (*r* = 0.27, 95% CI = 0.20, 0.34, *p* < 0.001; *I^2^* = 57.66%, *Q* = 34.07, *df* = 15, *p* < 0.01). The results were presented in a forest plot in Figure 6.

There was no significant variance within level 2 and level 3. Moderating effects of mean age, percentage of females, country, domains of functioning, and type of functioning measurements did not exist for the relationship between defusion and functioning.

**FIGURE 7:**
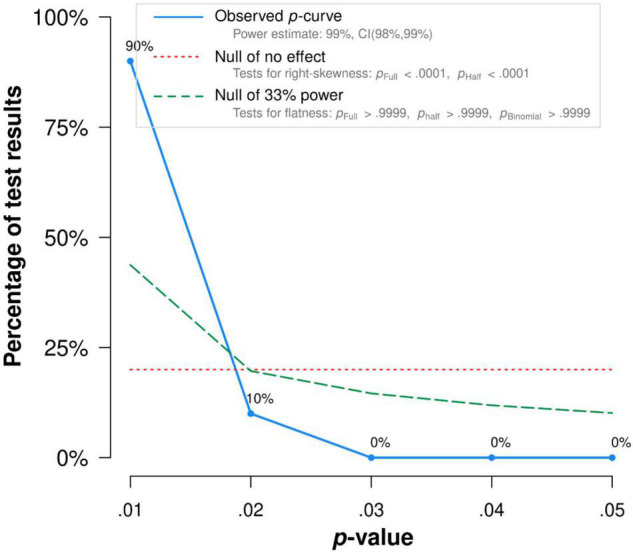
The *p*-Curve for statistically significant results on the relationship between defusion and functioning. The observed *p*-curve includes 10 statistically significant (*p* < 0.05) results, of which 10 are *p* < 0.025. There were 6 additional results entered but excluded from *p*-curve because they were *p* > 0.05.

There was no publication bias in Egger tests and Funnel plot (*p* > 0.1) on the relationship between acceptance and functioning. Both the full *p*-curve and the half *p*-curve test were significant with *p* < 0.0001 (*Z* = −10.82, *Z* = −10.32), which indicated that the distribution of *p-*values is significant right-skewed as seen in Figure 7. Hence, the results further support the initial assessment that evidential value is present in the literature.

#### Present Moment and Functioning

**FIGURE 8:**
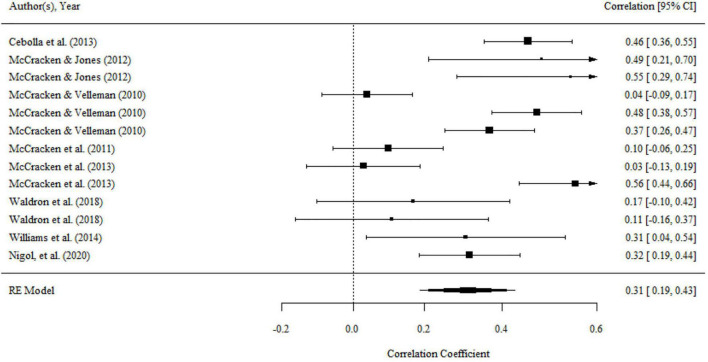
Forest plot of effect size (*r*) for the relationship between present moment and functioning.

There were 13 correlations in seven studies that examined the relationship between present moment and functioning. The overall effect size was statistically significant and medium (*r* = 0.31, 95% CI = 0.19, 0.43, *p* < 0.001; *I^2^* = 84.29%, *Q* = 79.70, *df* = 12, *p* < 0.001). The results were presented in a forest plot in Figure 8.

There was significant variance within the same studies (i.e., level 2 variance), while there was no significant variance between studies (i.e., level 3 variance). The details can be seen in Table 2. Moderator analyses were conducted in order to determine variables that can explain level 2 variance. We found a significant moderating effect of the domains of functioning on the association, as shown by the results of the omnibus test (*F*_(2,10)_ = 5.34, *p* < 0.05). The mean effect of the relationship between present moment and psychological functioning (*r* = 0.49, 95% CI = 0.29, 0.68, *p* < 0.001) was substantially larger than the association of present moment and physical functioning (*r* = 0.13, 95% CI = −0.08, 0.35, *p* > 0.05). No significant moderating effect was found for the percentage of females, mean age, country, and type of functioning measurements.

**FIGURE 9:**
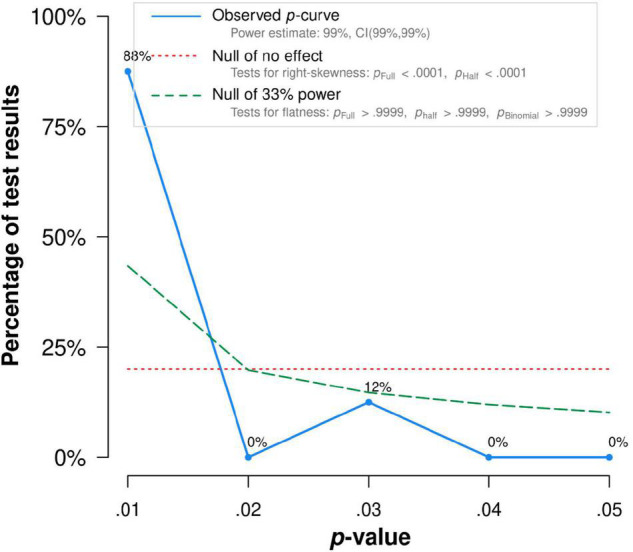
The *p*-Curve for statistically significant results on the relationship between present moment and functioning. The observed *p*-curve includes 8 statistically significant (*p* < 0.05) results, of which 8 are *p* < 0.025. There were five additional results entered but excluded from *p*-curve because they were *p* > 0.05.

There was no publication bias in Egger tests and Funnel plot (*p* > 0.1) on the relationship between the present moment and functioning. Both the full *p*-curve and the half *p*-curve test were significant with *p* < 0.0001 (*Z* = −12.54, *Z* = −11.52), which indicated that the distribution of *p*-values is significant right-skewed, as seen in Figure 9. Hence, the results further support the initial assessment that evidential value is present in the literature.

#### Self as Context and Functioning

**FIGURE 10:**
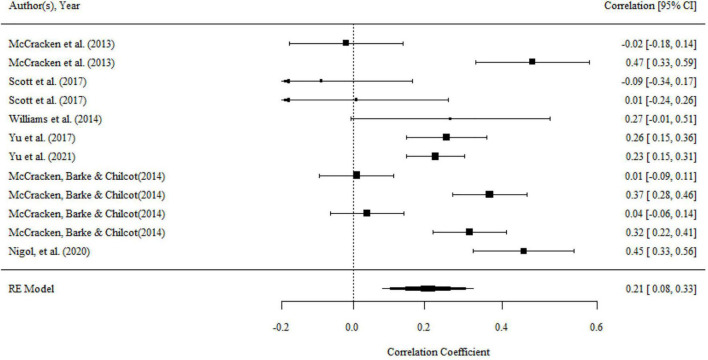
Forest plot of effect size (*r*) for the relationship between self as context and functioning.

There were 12 correlations in seven studies that examined the relationship between self as context and functioning. The overall effect size was significant (*r* = 0.21, 95% CI = 0.08, 0.33, *p* < 0.01; *I^2^* = 88.88%, *Q* = 83.72, *df* = 11, *p* < 0.001). The results were presented in a forest plot in Figure 10.

There was significant variance within the same studies (i.e., level 2 variance), while there was no significant variance between studies (i.e., level 3 variance). The details can be seen in Table 2. There was a significant moderating effect of the domain of functioning on the association, as shown by the results of the omnibus test (*F*_(2,10)_ = 29.56, *p* < 0.001). The mean effect of the relationship between self as context and overall functioning (*r* = 0.30, 95% CI = 0.19, 0.41, *p* < 0.001) and psychological functioning (*r* = 0.33, 95% CI = 0.19, 0.48, *p* < 0.001) was substantially larger than that association with physical functioning (*r* = −0.02, 95% CI = −0.17, 0.13, *p* > 0.05). No significant moderating effect was found for the percentage of females, mean age, country, type of functioning measurements.

**FIGURE 11:**
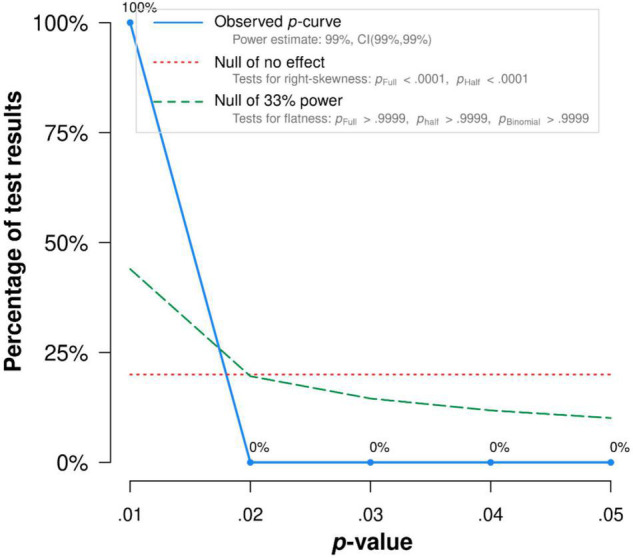
The *p*-Curve for statistically significant results on the relationship between self and context and functioning. The observed *p*-curve includes three statistically significant (*p* < 0.05) results, of which three are *p* < 0.025. There were four additional results entered but excluded from *p*-curve because they were *p* > 0.05.

There was no publication bias in Egger test, Funnel plot (*p* > 0.1), and trim-and-fill analyses. Both the full *p*-curve and the half *p*-curve test were significant with *p* < 0.0001 (*Z* = −13.15, *Z* = −12.83), which indicated that the distribution of *p*-values is significant right-skewed, as seen in Figure 11. Hence, the results further support the initial assessment that evidential value is present in the literature.

#### Values and Functioning

**FIGURE 12:**
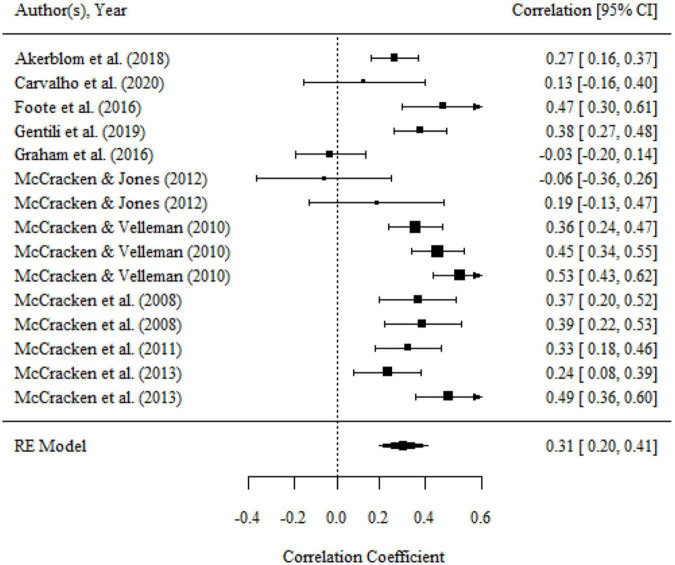
Forest plot of effect size (*r*) for the relationship between values and functioning.

There were fifteen correlations in ten studies that examined the relationship between values and functioning. The overall effect size was statistically significant and medium (*r* = 0.31, 95% CI = 0.20, 0.41, *p* < 0.01; *I^2^* = 79.78%, *Q* = 58.23, *df* = 15, *p* < 0.001). The results were presented in a forest plot in Figure 12.

There was no significant variance within the same studies (i.e., level 2 variance) and between studies (i.e., level 3 variance). The details can be seen in Table 2. We found a significant moderating effect of the domains of functioning on the association, as shown by the results of the omnibus test (*F*_(2,12)_ = 4.66, *p* < 0.05). The relationship between values and psychological functioning (*r* = 0.45, 95% CI = 0.34, 0.55, *p* < 0.05) was substantially larger than the association of values and physical functioning (*r* = 0.27, 95% CI = 0.04, 0.48, *p* < 0.05). No significant moderating effects were found for the percentage of females, mean age, country, and type of functioning measurements.

**FIGURE 13:**
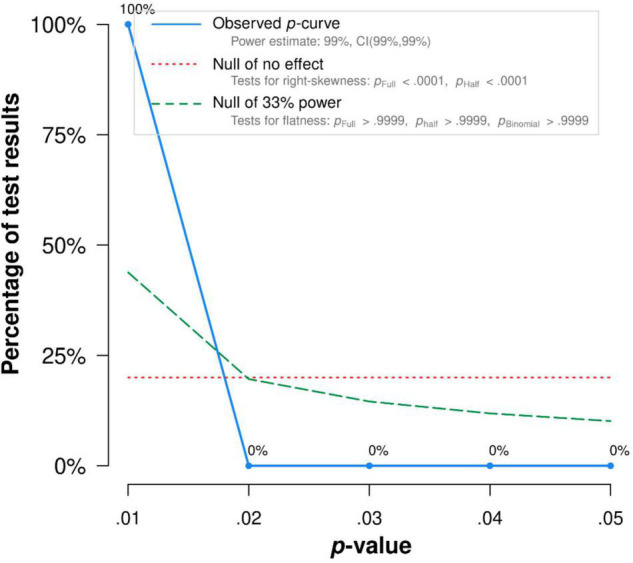
The *p*-Curve for statistically significant results on the relationship between values and functioning. The observed *p*-curve includes 11 statistically significant (*p* < 0.05) results, of which 11 are *p* < 0.025. There were four additional results entered but excluded from *p*-curve because they were *p* > 0.05.

There was no publication bias in Egger tests (*p* > 0.1) and the Funnel plot. Both the full *p*-curve and the half *p*-curve test were significant with *p* < 0.001 (*Z* = −15.41, *Z* = −14.85), which indicated that the distribution of *p*-values is significant right-skewed, as seen in Figure 13. Hence, the results further support the initial assessment that evidential value is present in the literature.

## Discussion

The present meta-analytic study aimed to estimate an overall association between functioning and six processes of psychological flexibility (i.e., acceptance, defusion, present moment, self as context, committed action, and values). A second aim was to assess whether the strength of these associations is influenced by domains of functioning, type of measurements of functioning, age of sample, country, and the proportion of females. In general, higher levels of psychological flexibility processes are significantly associated with higher levels of functioning. Except for the relationship between defusion and functioning, the relationships between the other five psychological flexibility processes and functioning were all moderated by domains of functioning. Specifically, the strength of the relationship between committed action and overall functioning exceeds its associations with psychological functioning. Also, the strength of the relationship between acceptance/self as context and overall functioning exceeds their associations with physical functioning. Besides, the mean effect of the relationship between acceptance/present moment/self as context/values and psychological functioning exceeds their associations with physical functioning. It was worth noting that the mean effect of the association between the present moment and physical functioning was not significant.

Acceptance is fostered as a behavioral response to pain and distress that cannot be directly changed to engage in meaningful but potentially painful activities (8). Thus, chronic pain patients with high acceptance could be more likely to accept their negative emotions and life events, and would not waste time on events or behaviors that are worthless, i.e., having high functioning. A prospective study found patients who reported greater acceptance at the base time would report better functioning in the future, which suggested that willingness to experience pain and accept it can lead to healthy functioning for chronic pain patients (51). In addition, acceptance is more related to overall and psychological functioning than physical functioning. It is not surprising, as there is a strong correlation between acceptance and an individual’s emotional or mental health (52, 53), and some scholars thought that acceptance alone is a better predictor of psychopathology and well-being than other variables (53). A randomized controlled trial found that acceptance and value clarification could improve participants’ social interaction after experiencing stressful social situations (54). The previous studies and present study both suggested that acceptance is strongly related to psychological functioning in patients with chronic pain. Enhancing personal acceptance may also mean enhancing individual psychological functioning. It may be due to the instruments used to measure overall functioning, such as the BPI (55), as the relationship between acceptance and overall functioning is also stronger than the association between acceptance and physical functioning. The BPI contains seven areas, including emotions and social and physical functioning, and emotions are closely associated with acceptance (52, 53).

Committed action is the ability to build and flexibly adhere to actions guided by values (56). As a behavior pattern oriented toward valued living, committed action may be important for the adaptive adjustment to pain in chronic pain patients (57, 58). Thus, if chronic pain patients have a high level of committed action, they may stay with the behavior or action that is useful to them (e.g., engaging in some recovery training or exercise) and would have a high level of physical functioning. In the present study, the relationship between committed action and physical functioning was higher than the association between committed action and psychological flexibility. However, there was no significant difference which may be due to the small number of studies included. Thus future studies should investigate whether there are differences between the two. The relationship between overall functioning and committed action was significantly higher than the association between psychological functioning and committed action, which may be due to the measurement of overall functioning aforementioned.

Also, cognitive defusion encourages patients to disengage or step back from thoughts and view them as what they are (i.e., merely cognitive events) rather than reality to reduce their impact on behavior (8). It was argued that defusion and acceptance loaded onto a single factor (58). Both processes relate to the willingness to deal with difficult experiences when attempts for change are ineffective or lead to further problems (58). Therefore, patients with greater defusion could lead to healthier functioning. Previous studies also suggested that chronic pain patients with a greater capacity to take a detached view of their own thoughts and emotional experiences (i.e., cognitive defusion) were more likely to suffer less and have better functioning (59).

McCracken and colleagues argued being more mindful or contacting the present moment can lead to a more “balanced, non-reactive and realistic” relationship to pain experiences (60). The present moment involves purposeful, non-judgmental, and fluid focus on present experiences (58, 61), which are not all directly related to pain but rather the processes of acceptance and the present moment play important roles in the suffering and functioning of chronic pain (60). Furthermore, chronic pain patients’ functioning can also be predicted by acceptance and present moment (60). Thus, the higher present moment is associated with higher functioning. However, in this study, we further found that physical functioning has a non-significant association with the present moment, while the present moment has a medium to large relationship with psychological functioning. A systematic review, which examined physical functioning and mindfulness skills training in chronic pain, suggested that contacting the present moment has no efficacy on physical functioning (62), while it has an important role in psychological functioning (63). These were consistent with our study.

In the present study, there was a small positive relationship between self as context and functioning. It should be noted that there was a small negative relationship between self as context and physiological function, but it was not significant. Self as context entails an experience of taking a perspective from their thoughts and feelings and distancing oneself from their thoughts and feelings, but it does not guide the patient’s behavior. Thus, self as context has a strong relationship with depression and can predict emotional functioning (28). And this may be the reason why the association between psychological/overall functioning and self as context were high than that with physical functioning.

Values can help chronic pain patients identify directions for meaningful activities essential to living (58). Treatment programs from ACT theoretical framework found that increased engagement in valued activities was significantly associated with greater improvement in psychological functioning but was not related to change in physical functioning at post-treatment (64). Thus, enhanced values orientation can have a more critical impact on psychological functioning than physical functioning. That may be why values have a higher relationship with psychological functioning in our analysis.

The hypothesized moderating effects (i.e., the percentage of females, mean age, country, and type of functioning measurements) were not found in this study, except for domains of functioning. The relatively narrow age range of participants in this study, which included only five studies that focused on adolescents, may limit detecting a real moderating effect of age on the associations. Also, most studies have focused on Europe and the United States, with very few studies on Asia (only one), so it would be difficult to identify the moderating role of culture. Therefore, future research in different cultural contexts is highly recommended, especially in Asia.

As no known studies have made a comprehensive meta-analysis of the relationship between specific processes of psychological flexibility (e.g., acceptance, defusion, present moment, self as context, committed action, and values) and different domains of functioning (i.e., physical functioning, psychological functioning, and overall functioning), the main strength of the current study lies in addressing this gap in the literature. The findings of this study produced more knowledge on the true associations between variables as well as contradictions or variances between studies. This study offers a possible explanation for why a particular therapy is more effective and can help researchers understand what is most important to pain patients and what might be more effective in improving their functioning. Knowing the relationship between the processes of psychological flexibility and functioning allows process-based therapy to be tailored to chronic pain patients. Patients can be better and more effectively served by emphasizing functioning-related psychological flexibility processes when designing intervention programs. The current study suggests that the ACT programs that focused on acceptance, committed action, the present moment, and values would be more recommendable or applicable to patients with chronic pain, because these processes have a medium to large relationship with functioning. Also, further study is needed to understand factors that influence functioning in attempting to mitigate functional impairment for chronic pain patients.

Although this study provided a conceptual and empirical basis for future work, there were some limitations. First, a major weakness of this meta-analysis is that the methodological quality of the studies was not rated. It was suggested that rating would be difficult due to the lack of clear methodological standards and relevant detail in the methods sections of these studies (56, 65). This study excluded unpublished studies, providing a general approach to ensure methodological quality, but also raising the risk of publication bias affecting the results. A more comprehensive search of the published and unpublished literature may be helpful for further research in this area. Second, the studies included were based on cross-sectional research, so the direction of causality remains unclear. Based on these findings, we cannot determine, for example, whether acceptance influences functioning, functioning influences acceptance, or (more likely) these factors have mutual influence. In addition, self-report data were used in included studies, which may lead to the inflationary effects of common method variance. Thus, the results need to be interpreted with caution. Longitudinal or experimental studies in which psychological flexibility processes are manipulated are needed to evaluate its potential causal impact on chronic pain patients’ functioning. Third, psychological flexibility and psychological inflexibility are two different concepts (50, 66) that were simplified in this study by reversing the results of measuring inflexibility to represent flexibility due to the limited number of available studies. Future research could explore the relationship between the different dimensions of psychological inflexibility and flexibility (i.e., the 12 dimensions) and domains of functioning. Besides, the current meta-analysis only examined gender, region, percentage of females, type of instruments, and domains of functioning as potential moderators. Other potential moderating variables (e.g., education level, family economic status) have not been analyzed and should be further explored in the future to investigate the role of other potential moderating variables in the relationship between functioning and psychological flexibility processes. Furthermore, region and culture are different, and the regional coding does not fully reflect the cultural context. Future research should explore a better way to code the cultural context. Finally, the number of some effect sizes of the moderation variables in the current meta-analytic studies are small, which may impact the results.

## Data Availability Statement

The original contributions presented in this study are included in the article/supplementary material, further inquiries can be directed to the corresponding author.

## Author Contributions

DD conceived and designed the analysis, collected the data, performed the analysis, and wrote the manuscript. MZ supported the data collecting, reviewed the included studies, and assisted in data analysis. Both authors contributed to the article and approved the submitted version.

## Conflict of Interest

The authors declare that the research was conducted in the absence of any commercial or financial relationships that could be construed as a potential conflict of interest.

## Publisher’s Note

All claims expressed in this article are solely those of the authors and do not necessarily represent those of their affiliated organizations, or those of the publisher, the editors and the reviewers. Any product that may be evaluated in this article, or claim that may be made by its manufacturer, is not guaranteed or endorsed by the publisher.

## References

[1] Brooks JM Iwanaga K Chiu CY Cotton BP Deiches J Morrison B Relationships between self-determination theory and theory of planned behavior applied to physical activity and exercise behavior in chronic pain. *Psychol Health Med.* (2017) 22:814–22. 10.1080/13548506.2017.1282161 28111983 PMC6013835

[2] Rhodes A Marks D Block-Lerner J Lomauro T. Psychological flexibility, pain characteristics and risk of opioid misuse in noncancerous chronic pain patients. *J Clin Psychol Med Settings.* (2021) 28:405–17. 10.1007/s10880-020-09729-1 32519037

[3] Si H Wang C Jin Y Tian X Qiao X Liu N Prevalence, factors, and health impacts of chronic pain among community-dwelling older adults in China. *Pain Manag Nurs.* (2019) 20:365–72. 10.1016/j.pmn.2019.01.006 31103518

[4] Esteve R Marcos E Reyes-Pérez Á López-Martínez AE Ramírez-Maestre C. Pain acceptance creates an emotional context that protects against the misuse of prescription opioids: a study in a sample of patients with chronic noncancer pain. *Int J Environ Res Public Health.* (2021) 18:3054. 10.3390/ijerph18063054 33809628 PMC8002364

[5] Goldbart A Bodner E Shrira A. The role of emotion covariation and psychological flexibility in coping with chronic physical pain: an integrative model. *Psychol Health.* (2020) 36:1299–313. 10.1080/08870446.2020.1841766 33136460

[6] Davis MC Zautra AJ Smith BW. Chronic pain, stress, and the dynamics of affective differentiation. *J Pers.* (2004) 72:1133–60. 10.1111/j.1467-6494.2004.00293.x 15509279 PMC2570251

[7] McCracken LM Vowles KE. Psychological flexibility and traditional pain management strategies in relation to patient functioning with chronic pain: an examination of a revised instrument. *J Pain.* (2007) 8:700–7. 10.1016/j.jpain.2007.04.008 17611162

[8] Wicksell RK Vowles KE. The role and function of acceptance and commitment therapy and behavioral flexibility in pain management. *Pain Manag.* (2015) 5:319–22. 10.2217/pmt.15.32 26238018

[9] Vowles KE Witkiewitz K Levell J Sowden G Ashworth J. Are reductions in pain intensity and pain-related distress necessary? An analysis of within-treatment change trajectories in relation to improved functioning following interdisciplinary acceptance and commitment therapy for adults with chronic pain. *J Consult Clin Psychol.* (2017) 85:87–98. 10.1037/ccp0000159 27991806

[10] Hayes S Hogan M Dowd H Doherty E O’Higgins S Gabhainn SN Comparing the clinical-effectiveness and cost-effectiveness of an internet-delivered Acceptance and Commitment Therapy (ACT) intervention with a waiting list control among adults with chronic pain: study protocol for a randomised controlled trial. *BMJ Open.* (2014) 4:e005092. 10.1136/bmjopen-2014-005092 24993763 PMC4091504

[11] Ding D Wang W. Psychological flexibility and job performance among it staff: a chained mediation model of workplace ostracism and perceived stress. *Psychologia.* (2022). 10.2117/psysoc.2021-A154 [Epub ahead of print].

[12] Kanstrup M Wicksell RK Kemani M Lipsker CW Lekander M Holmstrom L. A clinical pilot study of individual and group treatment for adolescents with chronic pain and their parents: effects of acceptance and commitment therapy on functioning. *Children.* (2016) 3:30. 10.3390/children3040030 27854323 PMC5184805

[13] Daks JS Peltz JS Rogge RD. Psychological flexibility and inflexibility as sources of resiliency and risk during a pandemic: modeling the cascade of COVID-19 stress on family systems with a contextual behavioral science lens. *J Contextual Behav Sci.* (2020) 18:16–27. 10.1016/j.jcbs.2020.08.003 32834972 PMC7428754

[14] Fang S Ding D. The efficacy of group-based acceptance and commitment therapy on psychological capital and school engagement: a pilot study among Chinese adolescents. *J Contextual Behav Sci.* (2020) 16:134–43. 10.1016/j.jcbs.2020.04.005

[15] Hayes SC Luoma JB Bond FW Masuda A Lillis J. Acceptance and commitment therapy: model, processes and outcomes. *Behav Res Ther.* (2006) 44:1–25. 10.1016/j.brat.2005.06.006 16300724

[16] McCracken LM Morley S. The psychological flexibility model: a basis for integration and progress in psychological approaches to chronic pain management. *J Pain.* (2014) 15:221–34. 10.1016/j.jpain.2013.10.014 24581630

[17] Brown SL Roush JF Marshall AJ Jones C Key C. The intervening roles of psychological inflexibility and functional impairment in the relation between cancer-related pain and psychological distress. *Int J Behav Med.* (2020) 27:100–7. 10.1007/s12529-019-09838-8 31898310

[18] Hughes LS Clark J Colclough JA Dale E McMillan D. Acceptance and commitment therapy (ACT) for chronic pain: a systematic review and meta-analyses. *Clin J Pain.* (2017) 33:552–68. 10.1097/ajp.0000000000000425 27479642

[19] Trindade IA Guiomar R Carvalho SA Duarte J Lapa T Menezes P Efficacy of online-based acceptance and commitment therapy for chronic pain: a systematic review and meta-analysis. *J Pain.* (2021) 22:1328–42. 10.1016/j.jpain.2021.04.003 33892153

[20] McCracken LM Barker E Chilcot J. Decentering, rumination, cognitive defusion, and psychological flexibility in people with chronic pain. *J Behav Med.* (2014) 37:1215–25. 10.1007/s10865-014-9570-9 24838420

[21] McCracken LM Velleman SC. Psychological flexibility in adults with chronic pain: a study of acceptance, mindfulness, and values-based action in primary care. *Pain.* (2010) 148:141–7. 10.1016/j.pain.2009.10.034 19945795

[22] McCracken LM Williams JL Tang NK. Psychological flexibility may reduce insomnia in persons with chronic pain: a preliminary retrospective study. *Pain Med.* (2011) 12:904–12. 10.1111/j.1526-4637.2011.01115.x 21539701

[23] Feinstein AB Forman EM Masuda A Cohen LL Herbert JD Moorthy LN Pain intensity, psychological inflexibility, and acceptance of pain as predictors of functioning in adolescents with juvenile idiopathic arthritis: a preliminary investigation. *J Clin Psychol Med Settings.* (2011) 18:291–8. 10.1007/s10880-011-9243-6 21630002

[24] Foote HW Hamer JD Roland MM Landy SR Smitherman TA. Psychological flexibility in migraine: a study of pain acceptance and values-based action. *Cephalalgia.* (2016) 36:317–24. 10.1177/0333102415590238 26063726

[25] Williams AM Cano A. Spousal mindfulness and social support in couples with chronic pain. *Clin J Pain.* (2014) 30:528–35. 10.1097/ajp.0000000000000009 24281274 PMC4013202

[26] Yu L Kioskli K McCracken LM. The psychological functioning in the COVID-19 pandemic and its association with psychological flexibility and broader functioning in people with chronic pain. *J Pain.* (2021) 22:926–39. 10.1016/j.jpain.2021.02.011 33677112 PMC7930808

[27] Scott W Daly A Yu L McCracken LM. Treatment of chronic pain for adults 65 and over: analyses of outcomes and changes in psychological flexibility following interdisciplinary acceptance and commitment therapy (ACT). *Pain Med.* (2017) 18:252–64. 10.1093/pm/pnw073 28204691

[28] Yu L Norton S Almarzooqi S McCracken LM. Preliminary investigation of self-as-context in people with fibromyalgia. *Br J Pain.* (2017) 11:134–43. 10.1177/2049463717708962 28785409 PMC5521349

[29] Wicksell RK Ahlqvist J Bring A Melin L Olsson GL. Can exposure and acceptance strategies improve functioning and life satisfaction in people with chronic pain and whiplash-associated disorders (WAD)? A randomized controlled trial. *Cogn Behav Ther.* (2008) 37:169–82. 10.1080/16506070802078970 18608312

[30] Wicksell RK Melin L Lekander M Olsson GL. Evaluating the effectiveness of exposure and acceptance strategies to improve functioning and quality of life in longstanding pediatric pain–a randomized controlled trial. *Pain.* (2009) 141:248–57. 10.1016/j.pain.2008.11.006 19108951

[31] Yu L Inspector Y McCracken LM. Preliminary investigation of the associations between psychological flexibility, symptoms and daily functioning in people with chronic abdominal pain. *Br J Pain.* (2021) 15:175–86. 10.1177/2049463720926559 34055339 PMC8138614

[32] Maathz P Flink IK Engman L Ekdahl J. Psychological inflexibility as a predictor of sexual functioning among women with vulvovaginal pain: a prospective investigation. *Pain Med.* (2020) 21:3596–602. 10.1093/pm/pnaa042 32186737 PMC7770233

[33] Bergner M Bobbitt RA Carter WB Gilson BS. The sickness impact profile: development and final revision of a health status measure. *Med Care.* (1981) 19:787–805. 7278416 10.1097/00005650-198108000-00001

[34] Gilpin HR Keyes A Stahl DR Greig R McCracken LM. Predictors of treatment outcome in contextual cognitive and behavioral therapies for chronic pain: a systematic review. *J Pain.* (2017) 18:1153–64. 10.1016/j.jpain.2017.04.003 28455249

[35] Hayes SC Hofmann SG. *Process-Based CBT: The Science and Core Clinical Competencies of Cognitive Behavioral Therapy.* Oakland, CA: New Harbinger (2018).

[36] Hofmann SG Hayes SC. Modern CBT: moving toward process-based therapies. *Rev Bras Ter Cogn.* (2018) 14:77–84. 10.5935/1808-5687.20180012

[37] Ferreira-Valente MA Pais-Ribeiro JL Jensen MP. Associations between psychosocial factors and pain intensity, physical functioning, and psychological functioning in patients with chronic pain: a cross-cultural comparison. *Clin J Pain*. (2014) 30:713–23. 10.1097/AJP.0000000000000027 24042349

[38] Page MJ McKenzie JE Bossuyt PM Boutron I Hoffmann TC Mulrow CD The PRISMA 2020 statement: an updated guideline for reporting systematic reviews. *J Clin Epidemiol.* (2021) 134:178–89. 10.1016/j.jclinepi.2021.03.001 33789819

[39] Cheung MW. Modeling dependent effect sizes with three-level meta-analyses: a structural equation modeling approach. *Psychol Methods.* (2014) 19:211–29. 10.1037/a0032968 23834422

[40] Gao S Assink M Cipriani A Lin K. Associations between rejection sensitivity and mental health outcomes: a meta-analytic review. *Clin Psychol Rev.* (2017) 57:59–74. 10.1016/j.cpr.2017.08.007 28841457

[41] Assink M Wibbelink CJM. Fitting three-level meta-analytic models in R: a step-by-step tutorial. *Quant Method Psychol.* (2016) 12:154–74. 10.20982/tqmp.12.3.p154

[42] R Core Team. *R: A Language and Environment for Statistical Computing.* Vienna: R Foundation for Statistical Computing (2016). https://www.r-project.org/

[43] Fernández-Castilla B. *Chapter 5_Appendix Publication Bias Three-Level*. (2019). Available online at: osf.io/2vkpz (accessed July 07, 2019).

[44] Lipsey MW Wilson D. *Practical Meta-Analysis.* Thousand Oaks, CA: Sage Publications (2001).

[45] Higgins JPT Green S. *Cochrane Handbook for Systematic Reviews of Interventions.* Hoboken, NJ: John Wiley & Sons Ltd (2008).

[46] Leandro G. *Meta-Analysis in Medical Research: The Handbook for the Understanding and Practice of Meta-Analysis.* Hoboken, NJ: Blackwell Publishing Ltd (2007). 10.1002/9780470994894

[47] Duval S Tweedie R. Trim and fill: a simple funnel-plot–based method of testing and adjusting for publication bias in meta-analysis. *Biometrics.* (2000) 56:455–63. 10.1111/j.0006-341X.2000.00455.x 10877304

[48] Simonsohn U Nelson LD Simmons JP. *p*-Curve and effect size: correcting for publication bias using only significant results. *Perspect Psychol Sci.* (2014) 9:666–81. 10.1177/1745691614553988 26186117

[49] Vogel D Homberg F. *P*-Hacking, *P*-curves, and the PSM–performance relationship: Is there evidential value?. *Public Adm Rev.* (2020) 81:191–204. 10.1111/puar.13273

[50] Daks JS Rogge RD. Examining the correlates of psychological flexibility in romantic relationship and family dynamics: a meta-analysis. *J Contextual Behav Sci.* (2020) 18:214–38. 10.1016/j.jcbs.2020.09.010

[51] McCracken LM Eccleston C. A prospective study of acceptance of pain and patient functioning with chronic pain. *Pain.* (2005) 118:164–9. 10.1016/j.pain.2005.08.015 16203093

[52] Elices M Tejedor R Pascual JC Carmona C Soriano J Soler J. Acceptance and present-moment awareness in psychiatric disorders: is mindfulness mood dependent?. *Psychiatry Res.* (2019) 273:363–8. 10.1016/j.psychres.2019.01.041 30682558

[53] Kotsou I Leys C Fossion P. Acceptance alone is a better predictor of psychopathology and well-being than emotional competence, emotion regulation and mindfulness. *J Affect Disord.* (2018) 226:142–5. 10.1016/j.jad.2017.09.047 28972931

[54] Hochard KD Hulbert-Williams L Ashcroft S McLoughlin S. Acceptance and values clarification versus cognitive restructuring and relaxation: a randomized controlled trial of ultra-brief non-expert-delivered coaching interventions for social resilience. *J Contextual Behav Sci.* (2021) 21:12–21. 10.1016/j.jcbs.2021.05.001

[55] Scott W Yu L Patel S McCracken LM. Measuring stigma in chronic pain: preliminary investigation of instrument psychometrics, correlates, and magnitude of change in a prospective cohort attending interdisciplinary treatment. *J Pain.* (2019) 20:1164–75. 10.1016/j.jpain.2019.03.011 30940501

[56] Levin ME Hildebrandt MJ Lillis J Hayes SC. The impact of treatment components suggested by the psychological flexibility model: a meta-analysis of laboratory-based component studies. *Behav Ther.* (2012) 43:741–56. 10.1016/j.beth.2012.05.003 23046777

[57] Galán S Roy R Solé E Racine M de la Vega R Jensen MP Committed action, disability and perceived health in individuals with fibromyalgia. *Behav Med.* (2019) 45:62–9. 10.1080/08964289.2018.1467370 29671691

[58] Vowles KE Sowden G Ashworth J. A comprehensive examination of the model underlying acceptance and commitment therapy for chronic pain. *Behav Ther.* (2014) 45:390–401. 10.1016/j.beth.2013.12.009 24680233

[59] McCracken LM Gutiérrez-Martínez O Smyth C. “Decentering” reflects psychological flexibility in people with chronic pain and correlates with their quality of functioning. *Health Psychol.* (2013) 32:820–3. 10.1037/a0028093 22545976

[60] McCracken LM Gauntlett-Gilbert J Vowles KE. The role of mindfulness in a contextual cognitive-behavioral analysis of chronic pain-related suffering and disability. *Pain.* (2007) 131:63–9. 10.1016/j.pain.2006.12.013 17257755

[61] Waldron SM Gauntlett-Gilbert J Marks E Loades ME Jacobs K. Dispositional mindfulness and its relationship with distress and functioning in adolescents with chronic pain and low-level pain. *J Pediatr Psychol.* (2018) 43:1038–46. 10.1093/jpepsy/jsy036 29800347

[62] Jackson W Zale EL Berman SJ Malacarne A Lapidow A Schatman ME Physical functioning and mindfulness skills training in chronic pain: a systematic review. *J Pain Res.* (2019) 12:179–89. 10.2147/JPR.S172733 30655687 PMC6322706

[63] Gu J Strauss C Bond R Cavanagh K. How do mindfulness-based cognitive therapy and mindfulness-based stress reduction improve mental health and wellbeing? A systematic review and meta-analysis of mediation studies. *Clin Psychol Rev.* (2015) 37:1–12. 10.1016/j.cpr.2015.01.006 25689576

[64] Vowles KE Sowden G Hickman J Ashworth J. An analysis of within-treatment change trajectories in valued activity in relation to treatment outcomes following interdisciplinary Acceptance and Commitment Therapy for adults with chronic pain. *Behav Res Ther.* (2019) 115:46–54. 10.1016/j.brat.2018.10.012 30409392

[65] Low CA Schauenburg H Dinger U. Self-criticism and psychotherapy outcome: a systematic review and meta-analysis. *Clin Psychol Rev.* (2020) 75:101808. 10.1016/j.cpr.2019.101808 31864153

[66] Rolffs JL Rogge RD Wilson KG. Disentangling components of flexibility *via* the hexaflex model: development and validation of the multidimensional psychological flexibility inventory (MPFI). *Assessment.* (2016) 25:458–82. 10.1177/1073191116645905 27152011

[67] Åkerblom S Perrin S Fischer M McCracken L. A validation and generality study of the committed action questionnaire in a Swedish sample with chronic pain. *Int J Behav Med.* (2016) 23:260–70. 10.1007/s12529-016-9539-x 26846475

[68] Åkerblom S Perrin S Rivano Fischer M McCracken LM. The relationship between posttraumatic stress disorder and chronic pain in people seeking treatment for chronic pain: the mediating role of psychological flexibility. *Clin J Pain.* (2018) 34:487–96. 10.1097/ajp.0000000000000561 29016388

[69] Beeckman M Hughes S Ryckeghem DV Hoecke EV Dehoorne J Joos R Resilience factors in children with juvenile idiopathic arthritis and their parents: the role of child and parent psychological flexibility. *Pain Med.* (2019) 20:1120–31. 10.1093/pm/pny181 30256982

[70] Carriere JS Sturgeon JA Yakobov E Kao M-C Mackey SC Darnall BD. The impact of perceived injustice on pain-related outcomes: a combined model examining the mediating roles of pain acceptance and anger in a chronic pain sample. *Clin J Pain.* (2018) 34:739–47. 10.1097/ajp.0000000000000602 29485535 PMC6424103

[71] Carvalho SA Pinto-Gouveia J Gillanders D Lapa T Valentim A Santos E Above and beyond emotional suffering: the unique contribution of compassionate and uncompassionate self-responding in chronic pain. *Scand J Pain.* (2020) 20:853–7. 10.1515/sjpain-2020-0082 32841171

[72] Catala P Suso-Ribera C Gutierrez L Perez S Lopez-Roig S Penacoba C. Is thought management a resource for functioning in women with fibromyalgia irrespective of pain levels? *Pain Med.* (2021) 22:1827–36. 10.1093/pm/pnab073 33595650

[73] Cebolla A Luciano JV DeMarzo MP Navarro-Gil M Campayo JG. Psychometric properties of the Spanish version of the Mindful Attention Awareness Scale (MAAS) in patients with fibromyalgia. *Health Qual Life Outcomes.* (2013) 11:6. 10.1186/1477-7525-11-6 23317306 PMC3554469

[74] Fish RA Hogan MJ Morrison TG Stewart I McGuire BE. Willing and able: a closer look at pain Willingness and Activity Engagement on the Chronic Pain Acceptance Questionnaire (CPAQ-8). *J Pain.* (2013) 14:233–45. 10.1016/j.jpain.2012.11.004 23452647

[75] Gauntlett-Gilbert J Alamire B Duggan GB. Pain acceptance in adolescents: development of a short form of the CPAQ-A. *J Pediatr Psychol.* (2019) 44:453–62. 10.1093/jpepsy/jsy090 30496433

[76] Gentili C Rickardsson J Zetterqvist V Simons LE Lekander M Wicksell RK. Psychological flexibility as a resilience factor in individuals with chronic pain. *Front Psychol.* (2019) 10:2016. 10.3389/fpsyg.2019.02016 31551871 PMC6734029

[77] Graham CD Gouick J Ferreira N Gillanders D. The influence of psychological flexibility on life satisfaction and mood in muscle disorders. *Rehabil Psychol.* (2016) 61:210–7. 10.1037/rep0000092 27196863

[78] Kanzler KE Pugh JA McGeary DD Hale WJ Mathias CW Kilpela LS Mitigating the effect of pain severity on activity and disability in patients with chronic pain: the crucial context of acceptance. *Pain Med.* (2019) 20:1509–18. 10.1093/pm/pny197 30590737 PMC6686120

[79] McCracken LM Zhao-O’Brien J. General psychological acceptance and chronic pain: there is more to accept than the pain itself. *Eur J Pain.* (2010) 14:170–5. 10.1016/j.ejpain.2009.03.004 19349199

[80] McCracken LM Jones R. Treatment for chronic pain for adults in the seventh and eighth decades of life: a preliminary study of Acceptance and Commitment Therapy (ACT). *Pain Med.* (2012) 13:860–7. 10.1111/j.1526-4637.2012.01407.x 22680627

[81] McCracken LM Vowles KE. A prospective analysis of acceptance of pain and values-based action in patients with chronic pain. *Health Psychol.* (2008) 27:215–20. 10.1037/0278-6133.27.2.215 18377140

[82] Nigol SH Di Benedetto M. The relationship between mindfulness facets, depression, pain severity and pain interference. *Psychol Health Med.* (2020) 25:53–63. 10.1080/13548506.2019.1619786 31124372

[83] Solé E Tomé-Pires C de la Vega R Racine M Castarlenas E Jensen MP Cognitive fusion and pain experience in young people. *Clin J Pain.* (2016) 32:602–8. 10.1097/ajp.0000000000000227 25803755

[84] Trainor H Henke M Winefield H Baranoff J. Functioning with fibromyalgia: the role of psychological flexibility and general psychological acceptance. *Aust Psychol.* (2019) 54:214–24. 10.1111/ap.12363

[85] Vasiliou VS Karekla M Michaelides MP Kasinopoulos O. Construct validity of the G-CPAQ and its mediating role in pain interference and adjustment. *Psychol Assess.* (2018) 30:220–30. 10.1037/pas0000467 28368172

[86] Wong CCY Paulus DJ Lemaire C Leonard A Sharp C Neighbors C Examining HIV-Related stigma in relation to pain interference and psychological inflexibility among persons living with HIV/AIDS: the role of anxiety sensitivity. *J HIV AIDS Soc Serv.* (2018) 17:1–15. 10.1080/15381501.2017.1370680 30034300 PMC6051718

[87] Yang SY McCracken LM Moss-Morris R. Psychological treatment needs for chronic pain in singapore and the relevance of the psychological flexibility model. *Pain Med.* (2017) 18:1679–94. 10.1093/pm/pnw175 27492743

[88] Zetterqvist V Holmström L Maathz P Wicksell RK. Pain avoidance predicts disability and depressive symptoms three years later in individuals with whiplash complaints. *Acta Anaesthesiol Scand.* (2017) 61:445–55. 10.1111/aas.12874 28233304

